# Human Papillomavirus 16 and 18 Infection in Oral Cancer in Thailand: A Multicenter Study

**DOI:** 10.31557/APJCP.2020.21.11.3349

**Published:** 2020-11

**Authors:** Nicha Komolmalai, Surawut Pongsiriwet, Nirush Lertprasertsuke, Suree Lekwanavijit, Sompid Kintarak, Ekarat Phattarataratip, Ajiravudh Subarnbhesaj, Kittipong Dhanuthai, Risa Chaisuparat, Anak Iamaroon

**Affiliations:** 1 *Department of Oral Biology and Diagnostic Sciences, Faculty of Dentistry, Chiang Mai University, Chiang Mai, Thailand. *; 2 *Department of Pathology, Faculty of Medicine, Chiang Mai University, Chiang Mai, Thailand.*; 3 *Department of Stomatology, Faculty of Dentistry, Prince of Songkla University, Songkhla, Thailand. *; 4 *Department of Oral Pathology, Faculty of Dentistry, Chulalongkorn University, Bangkok, Thailand. *; 5 *Department of Oral Diagnosis, Faculty of Dentistry, Khon Kaen University, Khon Kaen, Thailand. *

**Keywords:** HPV16/18, multicenter study, oral squamous cell carcinoma, p16, Thailand

## Abstract

**Objectives:**

To identify the prevalence of high-risk human papillomavirus (HPV) genotypes 16 and 18 among patients with oral squamous cell carcinoma (OSCC) in Thailand and investigate the associations of p16 expression and HPV16/18 with the demographic, clinicopathologic, and risk parameters.

**Materials and Methods:**

A total of 403 formalin-fixed paraffin-embedded OSCC specimens from four centers in four regions were obtained. p16 expression was evaluated by immunohistochemistry. The detection of HPV16/18 DNA was performed by polymerase chain reaction.

**Results:**

Of all, 172 specimens (42.7%) were presented with amplifiable extracted DNA. Among these, 62.8% were positive for p16, 8.1% were positive for HPV16/18, and 5.8% were positive for both methods. Of all HPV-positive specimens, HPV18 was detected in 57.1%; HPV16 in 14.3%; and HPV16 and 18 (co-infection) in 28.6%. The prevalence of HPV16/18 varied between centers, with the highest rate in the northern center (20.0%). There was no significant correlation between p16 expression and HPV16/18. There were no significant associations of p16 expression and/or HPV16/18 with all variables.

**Conclusions:**

The prevalence of HPV16/18 infection in OSCC geographically varied in Thailand, with the highest rate in the northern region. Poor correlation between p16 and HPV16/18 suggests p16 not be used as a surrogate marker for HPV-positive OSCC.

## Introduction

Oral squamous cell carcinoma (OSCC) is the most frequent histologic type, accounting for more than 90% of all oral cancers (Johnson et al., 2011). In Thailand, previous studies in the northern region showed an increasing rate of OSCC in the past two decades (Iamaroon et al., 2004; Komolmalai et al., 2015). Furthermore, the survival rate of Thai patients with OSCC was notably lower than those of the western countries (Pruegsanusak et al., 2012; Gatta et al., 2015; Chitapanarux et al., 2017). Conventionally, the major risk factors for OSCC include tobacco use, alcohol consumption, and betel quid chewing (IARC, 2012b). However, infection with high-risk human papillomavirus (HR-HPV) has been reported as an emerging risk factor for head and neck squamous cell carcinoma (HNSCC) (IARC, 2012a). 

HPVs are a heterogeneous group of small double-stranded, circular DNA viruses which can infect epithelial cells of the skin and mucosa (Burd, 2016; Sano and Oridate, 2016). Until now, more than 150 types of HPV are identified and categorized into low-risk and high-risk based on their malignant potential (Sano and Oridate, 2016). LR-HPVs such as types 6, 11, 40, 42, 43, 44, 54, 61, 72, 81, 89 were associated with anogenital warts and other benign lesions (Burd, 2016). On the other hand, HR-HPVs such as types 16, 18, 31, 33, 34, 35, 39, 45, 51, 52, 56, 58, 59, 66, 68, and 70 were frequently detected in cervical cancer and precancerous lesions (Sano and Oridate, 2016). Moreover, infection with HR-HPVs was found to be associated with cancers at the other sites, including vulva, vagina, penis, anus, oropharynx, and oral cavity (IARC, 2012a). Upon infection, E6 and E7 proteins of the HR-HPVs can cause degradation of p53 protein and inhibit the function of retinoblastoma protein (pRb), causing defects in apoptosis and DNA damage repair, cell cycle dysregulation, and cell immortalization. In addition, the pRb inhibition by E7 protein can induce an upregulation of p16INK4a (p16) protein, hence p16 expression suggested as a surrogate marker for HPV infection in tissues (Burd, 2016; Sano and Oridate, 2016). 

In OSCC, the prevalence of HPV infection was 24.2% globally (Ndiaye et al., 2014). The most frequent types were HPV16 and 18, while the other HR-HPVs were rarely detected (less than 1%) (Kreimer et al., 2005; Ndiaye et al., 2014). Individuals with HPV infection had a 4.40-fold increased risk of developing OSCC (Saulle et al., 2015). The HPV prevalence in OSCC varied between geographic regions, with the highest rates among Asian countries (Kreimer et al., 2005; Ndiaye et al., 2014). In Thailand, various results of HPV prevalence (0-57%) have been reported (Khovidhunkit et al., 2008; Sritippho et al., 2016; Chuerduangphui et al., 2017; Phusingha et al., 2017; Chotipanich et al., 2018). 

The distinction between HPV-related and -unrelated HNSCC has been established. The patients with HPV-positive HNSCCs tended to be younger, white, men, and individuals with local T category and poorly differentiated tumors (Li et al., 2018). Sexual behaviors such as oral sex practice and having multiple lifetime number of oral or genital sexual partners were associated with these cases (Emmett et al., 2018; Laprise et al., 2019). Regarding the prognosis, a significantly better survival and treatment response were observed among those with HPV infection (Li et al., 2018; Tian et al., 2019). 

To date, three prophylactic HPV vaccines including Cervarix^TM^ (bivalent), Gardasil^®^ (quadrivalent), and Gardasil®9 (nonavalent) were approved by the Food and Drug Administration (Takes et al., 2015). HPV vaccination for girls and young women was implemented to prevent cervical cancer in many countries (Herrero et al., 2015; Takes et al., 2015). Some countries such as Australia, Canada, and United States also recommended HPV vaccination in males to prevent anogenital warts and anal cancer (Stanley, 2014; Takes et al., 2015). Considering the higher rate of HPV-related HNSCC among men, HPV vaccination in not only girls, but also boys would be of great importance.

The purposes of this study were to identify the prevalence of HPV types 16 and 18 infection among patients with OSCC in Thailand; and to investigate the associations of p16 expression and HPV16/18 with the demographic, clinicopathologic, and classic risk parameters.

## Materials and Methods

*Specimen collection*

A total of 403 formalin-fixed, paraffin-embedded (FFPE) OSCC specimens, collected during January 1999 to January 2019, were received from archives of the Oral Pathology Laboratories, Faculties of Dentistry, Chiang Mai University (CMU, northern region, n=103); Prince of Songkla University (PSU, southern region, n=99); Chulalongkorn University (CU, central region, n=108); and Khon Kaen University (KKU, northeastern region n=93). Ethical clearances were obtained as follows: CMU (no. 11/2018), PSU (EC6010-31-L-LR), CU (HREC-DCU 2018-005), and KKU (HE612246). All specimens were primary tumors, histologically diagnosed with conventional OSCC or oral verrucous carcinoma (OVC). Age at diagnosis, gender, site of tumor, clinical tumor size, histologic grade; and the history of three classic risk behaviors including tobacco smoking, alcohol consumption, and betel quid chewing were collected from the laboratory records. The histopathologic diagnosis and grade of all specimens were re-evaluated by an experienced pathologist (NL) using the World Health Organization classification (Barnes et al., 2005).

*p16 immunohistochemistry (IHC)*

A 3-µm thick section was cut and p16 IHC was performed with the Ventana Benchmark ULTRA autostainer (Ventana Medical Systems, Tucson, AZ, USA) using the CINtec® p16 Histology Kit (clone E6H4, MTM/Roche laboratories AG, Heidelberg, Germany). A positive control of cervical carcinoma was included. p16 immunostaining was examined under a light microscope and scored by an experienced pathologist and an investigator with consensus agreement. The scoring was performed using a 5-tiered system as described by Prigge et al., 2015: none of the tumor cells are stained (-, negative); positive staining in 1-9% (+/-); 10-49% (1+); 50-89% (2+); and ≥90% (3+) of the tumor cells. The kappa scores of 0.87 (95% CI 0.81-0.93) and 0.80 (95% CI 0.73-0.87) were evident for intra-observer and inter-observer agreement of p16 scoring, respectively. The specimens with any staining above the background in the invasive parts of tumor were considered p16-positive (Smeets et al., 2007). 

*HPV16 and 18 DNA detection by polymerase chain reaction (PCR)*

Genomic DNA was extracted using the QIAamp^®^ DNA FFPE Tissue Kit (Qiagen GmbH, Hilden, Germany) and subsequently assessed for quantity and purity by the NanoDropTM 2000 Spectrophotometer (Thermo Fisher Scientific, Wilmington, DE, USA). PCR amplification of the house-keeping beta-actin gene was performed to confirm the integrity of extracted DNA and the specimens with negative results were excluded. The amplification of HPV16 and 18 DNA was then performed using the forward and reverse primers specific to the E6 gene as described in a previously study (Sritippho et al., 2016). A 25-μl PCR reaction contained 1× PCR buffer, 0.2 mM dNTP mixture, 1.5 mM MgCl_2_, 0.2 µM of each primer, 1 unit of PlatinumTM Taq DNA polymerase (InvitrogenTM, Life Technologies, São Paulo, Brazil), and 5 µl of DNA template. DNA extracted from SiHa and HeLa cell lines was included as a positive control for HPV16 and HPV18, respectively. A negative control was included in each run. The PCR conditions were 95°C for 5 min, 45 cycles of 94°C for 30 s, 55°C for 30 s, and 72°C for 30 s, and a final extension at 72°C for 7 min. The PCR products were analyzed by electrophoresis on 1.5% agarose gel and photographed using the ChemiDocTM Touch Imaging System (Bio-Rad Laboratories, Hercules, CA, USA).

*Statistical analysis*

The statistical analysis was performed using the IBM® SPSS® Statistics version 23. The Cohen’s kappa test was used to evaluate the concordance between p16 IHC and HPV16/18 PCR. The Chi-square or Fisher’s exact test was used to analyze the associations of p16 expression and HPV16/18 with age category, sex, site of tumor, clinical tumor size, histopathologic variant and grade, and the risk behaviors. The results with p value less than 0.05 were considered statistically significant. 

## Results

Of all, DNAs extracted from 172 specimens (42.7%) were positive for beta-actin gene and further analyzed. The median age at diagnosis of all patients was 66. The male-to-female ratio was 1:1.2. The demographic, clinicopathologic, and risk factor data for all cases are shown in Table 1.

**Figure 1:**
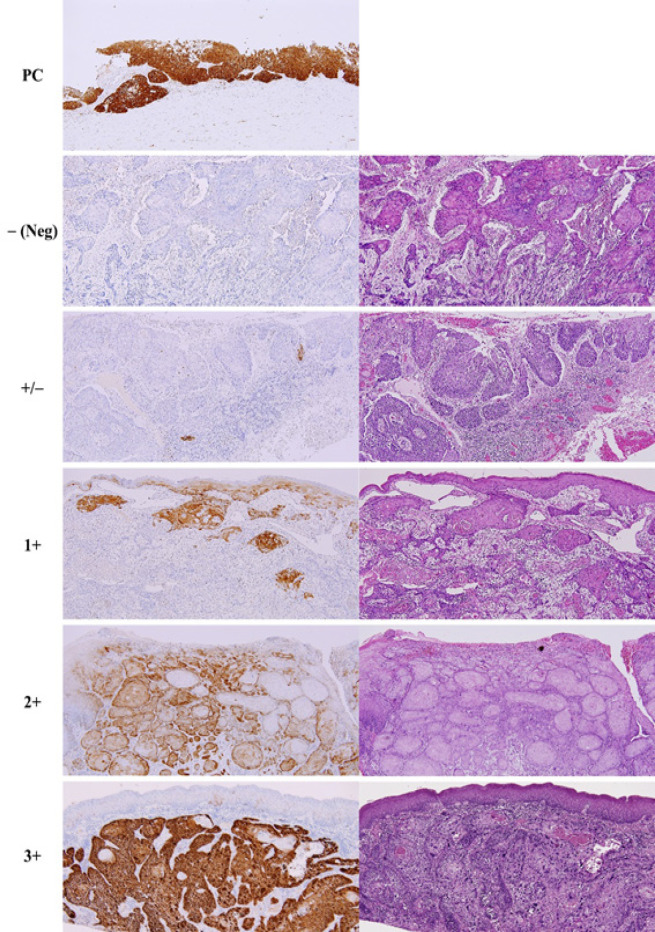
p16 Expression in Cervical Carcinoma (Positive Control, PC) and p16 Scoring in Representative OSCC Specimens. Left panel: p16 IHC; right panel: H&E, ×100 original magnification

**Figure 2:**
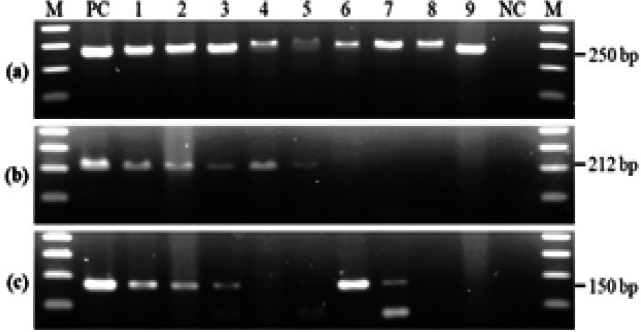
PCR Amplifications of Beta-Actin (a), HPV16 E6 (b), and HPV18 E6 (c) Genes. Lane M: 100-bp DNA ladder marker; lane PC: positive control; lanes 1-3: HPV16 and 18-positive specimens; lanes 4 and 5: HPV16-positive, HPV18-negative specimens; lanes 6 and 7, HPV16-negative, HPV18-positive specimens; lanes 8 and 9: HPV16 and 18-negative specimens; lane NC: negative control

The p16 scoring in representative examples is displayed in Figure 1. Of all 172 specimens, 62.8% showed p16 staining in the tumor cells. For all centers, HPV16 and/or 18 DNA was detected in 14 specimens (8.1%). Among these, 2 (14.3%) were positive for HPV16, 8 (57.1%) were positive for HPV18, and 4 (28.6%) were positive for both HPV16 and HPV18 (co-infection). Regarding each center, HPV16/18 was found in the specimens from the northern center (20.0%) and the southern center (6.9%), while it was not detected in those from the central and northeastern centers. The PCR products of representative cases are shown in Figure 2. The PCR protocols in this study were validated using serially diluted DNA extracted from SiHa and HeLa cell lines and yielded detection limits of 6 copies/µl for HPV16 and 322 copies/µl for HPV18.

The findings by both assays are summarized in Table 2. Of all specimens, 5.8% were positive for both methods. The concordant between the two assays was poor (κ=0.023, p=0.485). There were no significant associations of p16 and/or HPV16/18 with the demographic, clinicopathologic, and risk factor variables (Table 1). 

**Table 1:** Demographic, Clinicopathologic, and Risk Behavior Data and Their Associations with p16 Expression and HPV16/18 DNA

Variables	No. cases		p16 IHC+			HPV16/18 DNA+			p16 IHC+ and HPV16/18 DNA+		
	(N=172)										
	n	(%)	n	(%+)	p†	n	(%+)	p†	n	(%+)	p†
Age category (year)					0.301			0.145			0.669
<50	24	14	17	70.8		1	4.2		1	4.2	
50-69	90	52.3	59	65.6		11	12.2		7	7.8	
≥70	58	33.7	32	55.2		2	3.4		2	3.4	
Gender					0.338			0.845			0.515
Male	78	45.3	52	66.7		6	7.7		6	7.7	
Female	94	54.7	56	59.6		8	8.5		4	4.3	
Site					0.509			0.395			0.186
Tongue	46	26.7	28	60.9		6	13		5	10.9	
Gum/alveolar mucosa	70	40.7	44	62.9		5	7.1		3	4.3	
Buccal/labial mucosa	21	12.2	10	47.6		1	4.8		0	0	
Floor of mouth	8	4.7	6	75		1	12.5		1	12.5	
Retromolar area	7	4.1	6	85.7		1	14.3		1	14.3	
Other sites	20	11.6	14	70		0	0		0	0	
Clinical tumor size (cm)					0.566‡			0.441‡			0.302‡
≤2	49	28.5	29	59.2		2	4.1		1	2	
>2 and ≤4	34	19.8	24	70.6		4	11.8		3	8.8	
>4	16	9.3	10	62.5		1	6.3		1	6.3	
Missing data	73	42.4									
Histologic characteristics					0.374			0.725			0.474
MIC SCC	9	5.2	7	77.8		0	0		0	0	
WD SCC	127	73.8	74	58.3		11	8.7		7	5.5	
MD SCC	22	12.8	17	77.3		2	9.1		2	9.1	
PD SCC	6	3.5	4	66.7		1	16.7		1	16.7	
VC	8	4.7	6	75		0	0		0	0	
Tobacco smoking					0.108‡			>0.999‡			0.643‡
Ever	26	15.1	22	84.6		5	19.2		5	19.2	
Never	12	7	7	58.3		2	16.7		1	8.3	
Missing data	134	77.9									
Alcohol consumption					>0.999‡			0.591‡			>0.999‡
Ever	9	5.2	6	66.7		1	11.1		1	11.1	
Never	11	6.4	7	63.6		3	27.3		2	18.2	
Missing data	152	88.4									
Betel quid chewing					>0.999‡			0.609‡			0.544‡
Ever	13	7.6	8	61.5		2	15.4		1	7.7	
Never	9	5.2	6	66.7		3	33.3		2	22.2	
Missing data	150	87.2									

MD, moderately differentiated; MIC, microinvasive; PD, poorly differentiated; SCC, squamous cell carcinoma; VC, verrucous carcinoma; WD, well differentiated; †, Chi-square or Fisher’s exact test; ‡, The cases with missing data were excluded.

**Table 2:** Cross-Tabulation Table of p16 Expression by IHC and HPV16/18 Detection by PCR in the OSCC Specimens

	HPV16/18 DNA+	HPV16/18 DNA-	Total
p16+	10	98	108
p16-	4	60	64
Total	14	158	172

## Discussion

Globally, the pooled HPV prevalence in OSCC ranged from 16.9 to 24.2% (Kreimer et al., 2005; Ndiaye et al., 2014; Saulle et al., 2015). In this study, the prevalence of HPV16 and/or 18 was 8.1%. When compared with previous studies in Thailand, the rate is similar to a study (7.7%) (Chotipanich et al., 2018), but lower than other studies (15.8%-57%) (Sritippho et al., 2016; Chuerduangphui et al., 2017; Phusingha et al., 2017). The discrepancy between studies may reflect the differences in specimen selection, methods of HPV detection, HPV genotypes inclusion, and trend of risk behaviors. 

Regarding HPV subtypes, most studies reported that HPV16 was the most predominant type in OSCC, followed by HPV18 (Kreimer et al., 2005; Ndiaye et al., 2014). On the contrary, the prevalence of HPV18 in this study was higher than that of HPV16. Nevertheless, a higher prevalence of HPV18 in OSCC cases in relation to HPV16 was also reported among some studies in South Asian countries (Kulkarni et al., 2011; Zil et al., 2018). 

When only OVC cases is considered, the HR-HPV prevalence in previous studies ranged from 14.3 to 47.8% (Fujita et al., 2008; Saghravanian et al., 2015; Sritippho et al., 2016). However, we did not detect HPV16/18 among the OVC cases, which may be accounted by the limited numbers of these specimens. Moreover, other HR-HPVs previously detected in VCs such as types 33, 35, and 45 (Fujita et al., 2008; del Pino et al., 2012) were excluded. 

Among centers, the highest prevalence of HPV16/18 was observed in the northern center. We did not detect HPV16/18 in the specimens from central and northeastern centers, which is in contrast with some reports from these two regions (Khovidhunkit et al., 2008; Chuerduangphui et al., 2017; Phusingha et al., 2017; Chotipanich et al., 2018). There are several possible explanations for the discrepant rates between centers. The differences in method and period of specimen collection may affect the rate of DNA degradation. The demographic, clinicopathologic, and risk behavior variables were also varied among centers. Moreover, our results may reflect the general prevalence of HPV infection in each region. According to a multicenter study, the highest incidence of cervical cancer was observed among northern population (Bray et al., 2017). Another study reported the highest prevalence of cervical HPV infection in southern region (97.1%), followed by northern region (91.3%), northeastern region (87.9%), and the lowest in the central region (78.6%) (Suthipintawong et al., 2011). 

p16 IHC is widely used as an indirect marker for transcriptionally active-HPV due to its high sensitivity and feasibility with FFPE materials (Prigge et al., 2017). In 2018, the College of American Pathologists recommended the use of p16 IHC as a surrogate marker for HR-HPV testing in all new patients diagnosed with OPSCC, while for patients with non-oropharyngeal HNSCC, such testing should not be included in the routine practice (Lewis et al., 2018). In this study, the concordance between p16 expression and HPV16/18 was poor, which is similar to some previous studies (Ramshankar et al., 2014; Zafereo et al., 2016; Minami et al., 2017). Therefore, p16 may not be appropriate as a surrogate marker for HR-HPV infection in OSCCs. In addition, discrepant results between these two methods were also noted in our study. We found that 28.6% of all HPV16/18-positive specimens did not show any p16 staining in the tumor cells, which may imply a bystander HPV16/18 infection or an active HPV infection without p16 upregulation. The latter assumption was also seen in some previous studies which reported a subset of HNSCC cases with HPV E6/E7 mRNA-positive but lack of p16 expression (Hoffmann et al., 2012; Minami et al., 2017). Conversely, among all HPV16/18-negative specimens, p16 expression was observed in 62.0%. Infection with other HR-HPV types and an upregulation of p16 by alternate pathways may be attributable for this result. Nevertheless, an upregulation of p16 independent of HPV infection has also been reported in a subset of HNSCCs (Sgaramella et al., 2015; Dreyer et al., 2017).

Regarding the patient profiles, our results demonstrated no significant associations between HPV status and all parameters, which are in line with some studies (Duray et al., 2012; Kruger et al., 2014; de Abreu et al., 2018). On the contrary, other studies reported significantly higher rates of HPV infection among younger patients (Zhang et al., 2004; Sritippho et al., 2016); male patients (Zhang et al., 2004; Awan et al., 2017); tumors at the tongue (Phusingha et al., 2017); early TNM stage (stage I and II) and poorly differentiated tumors (Zhao et al., 2009; Lingen et al., 2013); and those with no history of tobacco smoking (Zhao et al., 2009) and no oral (smokeless) tobacco usage (Kane et al., 2015).

There are some limitations in the present study. First, the wide range of year collection of specimens could affect the quality of DNA. Second, since all data were collected from the history records; thus, there were several missing data regarding the clinical tumor size and the classic risk behaviors. Therefore, these may compromise the analyses of the clinical and risk behavior profiles.

In conclusion, the prevalence of HPV16/18 infection in OSCC varied between different regions in Thailand, with the highest rate among the northern population. The prevalence of HPV18 was twice higher than that of HPV16. The concordance between these two methods was poor. Further studies are needed to establish the association between HPV infection and sexual behaviors among Thai population. Moreover, the difference in survival outcome of Thai patients with OSCC regarding their HPV status requires further investigation.
